# Getting Beyond the Toy Domain. Meditations on David Deamer’s “Assembling Life”

**DOI:** 10.3390/life10020018

**Published:** 2020-02-18

**Authors:** William Bains

**Affiliations:** 1 Five Alarm Bio Ltd., O2h Scitech Park, Mill Lane, Hauxton, Cambridge CB22 5HX, UK; william@williambains.co.uk; 2 Department of Earth, Atmospheric and Planetary Sciences, Massachusetts Institute of Technology, Cambridge, MA 02139, USA

**Keywords:** origin of life, hydrothermal, alkaline vent, Copernican principle, toy domain

## Abstract

David Deamer has written another book, *Assembling Life,* on the origin of life. It is unapologetically polemic, presenting Deamer’s view that life originated in fresh water hydrothermal fields on volcanic islands on early Earth, arguing that this provided a unique environment not just for organic chemistry but for the self-assembling structure that drive that chemistry and form the basis of structure in life. It is worth reading, it is an advance in the field, but is it convincing? I argue that the Origin of Life field as a whole is unconvincing, generating results in Toy Domains that cannot be scaled to any real world scenario. I suggest that, by analogy with the history of artificial intelligence and solar astronomy, we need much more scale, and fundamentally new ideas, to take the field forward.

David Deamer has written another book on the origin of life (*Assembling Life—*hereafter AL) [1], and it starts with a near apology for why he wrote another when his last, semi-popular book (*First Life—*hereafter FL) covered the ground extremely well [2]. AL is more scholarly, with references and more detailed arguments, and more chemistry. FL was more of an overview, albeit coloured by Deamer’s own ideas. AL is unapologetically polemic, presenting Deamer’s view that life originated in fresh water hydrothermal fields on volcanic islands on early Earth. He is explicit that this is a conjecture, at most a hypothesis. Is it convincing?

To signal my intentions from the start, yes, I think you should read both books. FL is fun, a cracking read, detailed and thorough. It is also full of David’s trademark personal asides and comments, especially on the origin of the words and hence on their semantic baggage. AL also has trademark Deamer touches, as well as great introductions to background concepts. So read both, because Deamer has done a good job of laying out all the more mainstream options, and developing his thesis that life probably originated in fresh water hydrothermal systems from self-organizing fatty acid lamellae. I find the argument compelling to the extent that it fills in some of the gaps.

Deamer’s view might be summarised as “container-enabled chemistry first”. Long chain fatty acids can be made abiotically, and spontaneously assemble into multilayer structures on drying from freshwater hydrothermal fields, structures that break up again into vesicles on wetting. Why fresh water hydrothermal systems? Because in seawater divalent cations, especially calcium, prevent fatty acids from forming bilayers, as well as efficiently precipitating phosphate and organophosphate compounds (although the role of phosphate in OOL is itself contentious [3]). Other features Deamer cites as important to subaerial hydrothermal fields are frequent wet/dry cycles, both to form multilamellar structures and drive dehydration reactions, a variety of gradients of pH and temperature, ready recycling of input chemicals, chemistry that mobilizes all the major elements of biochemistry, air–water and air–rock as well as water–rock interfaces, availability of light to drive chemistry, and weathering to provide clays.

But the importance of this environment for Deamer lies not just in the chemical reactions it enables. Rather, it is its ability to enable self-assembly, which he considers primary and fundamental. Hence the title of the book, and the order of the chapters—Self-assembly, *then* macromolecules, *then* chemistry. His view is that there are a range of sources of organics in all scenarios of prebiotic Earth, and the problem to be solved is how they organize, both through self-assembly and polymerization. After some good introductions to what we know (very little), can reliably model (some) and conjecture (a lot) about the early Earth, he gets down to business in Chapter 4, all of which is devoted to potential sources of organics. Some sources (such as schemes that require Fischer–Tropsch synthesis of fatty acids over sulphide minerals) are hard to reconcile with a hydrothermal field environment, but we pass on. Chapter 5 is boldly titled “Self-assembly processes were essential for life’s origins”, and this is the core of his thesis. Not only does self-assembly create physical structures, it creates chemical environments that can enable early synthesis. Some of this is over-stated. Yes, DNA will spontaneously form double helices, but only if incubated at the appropriate temperature in the right ions, and you need to incubate for days to get half a bacterial genome to re-associate even at mg/mL concentrations [4,5]. Yes, some evolved enzymes fold spontaneously, but many do not (they need the chaperone machinery in cells to fold, and even then they sometimes fail to fold properly [6,7]), and even evolved proteins have a tendency to aggregate and precipitate if their environment is changed just a little, as Alzheimer’s Disease among others shows [8]. This sort of mess is just ignored in diagrams that treat chemicals as geometric shapes and nucleic acids as lines.

Deamer has at least part of the solution to how functional, or at least stable, polymers could be found among the vast majority of non-functional ones (putting side for a moment what we mean by “function”). Cycling fatty acids between dehydrated and hydrated states in fresh water allows for the generation of a huge population of self-organizing vesicles, each of which will be slightly different. Dehydration also drives condensation reactions. Of billions of vesicles formed, *if* a few accumulate random polymers and *if* those random polymers contribute to the vesicle’s stability, then the polymers and their lipid components are preserved. (They are not “replicated” because there is no information transfer). This sort of random combinatorics could select a combination of properties through cycling through vesicle, gel and lamella phases of the fatty acids, a process Damer and Deamer call “coupled phases” [9]. So the millions-to-one chance that a polymer with useful physical properties and chemical function is generated abiotically is overcome by millions of experiments. (Unsolved is how that combination of lipids and polymers remain associated during the dehydration stage, but—small steps.).

Unusually, Deamer devotes all of Chapter 9 to actual lab results. For example, he actually *shows* that fatty acids spontaneously form multilammellar structures on drying, in lab and in actual hydrothermal water samples. He has demonstrated this with organics extracted from the Murchison meteorite, so this works with real abiotic samples, not just lab chemicals. He shows that lamella formation drives the formation of something like RNA from nucleoside phosphates, a specific example of the hypothesised synthesis and subsequent kinetic trapping of dehydration polymers by wet/dry cycles working on monomers trapped between lipid layers, and that these are retained inside smaller, vesicle-like structure when the lamellae are wetted again. He has gone to actual, real hydrothermal ponds and dropped chemicals into them to see what will happen, pools as extreme as pH = 0 vent water, and shown dehydration polymerization under pool conditions [10]. This is moving well beyond geometric shapes and lines.

Chapter 10, co-authored with Deamer’s collaborator Bruce Damer, then pulls all the arguments together. It starts with a bold statement: “This chapter proposes that life can begin with chance ensembles of encapsulated polymers, *some of which happen to encode genetic information in the linear sequence of their monomers while other catalyse polymerization reactions*” (my italics). The chapter then goes through the steps, the evidence, and future tests. This has been expanded in [9,11], and in both book and papers Deamer both summarises the data supporting the idea and proposes future experiments to test it.

Deamer suggests that the next step is to go beyond one-at-a-time chemistry and try a wider range of chemistries, almost a Miller–Urey-type experiment but looking for structure, not chemistry. His Figure 8.1 (AL) shows his system for doing this in 24 combinations of environments at once. In this, he is aligned with Smith and Morowitz’ comment that “Rather than seeking the emergence of a novel and unified form of order from the beginning … we seek the dispersed opportunities for chemical and physical order provided by the Earth’s chemistry and dynamics, and ask how they were brought into interdependence and thus granted partial autonomy from non-living geospheres” [12]. Deamer’s multilamellar systems are not just abiotic chemistry, they are abiotic order. This is a bold vision, and one that I believe takes the field forward.

But. 

Here is the fundamental problem. There is only one actual fact known about the origin of life (OOL). It happened. We do not know where, when, or how. Suggestions on location range over clouds [13,14,15], in ice [16], black smokers [17,18], alkaline hydrothermal vents [19,20,21], freshwater volcanic [22] or sedimentary [23] hydrothermal systems, land ponds, coastal ponds, the Moon [24] and Mars [25]. Unique mineral requirements include sulphides [26] or green rust [20] or borates [27], or silica gels [23] or clays [18,28]. (Robert Hazen elegantly gets round this by pointing out that real minerals are complex mixes, and so you can have all of those in one rock [29,30]). The first container was lipid, or protein, or mineral microchannels, or life did not need a container because the chemistry was inherently confined to the surface of clays [31] or other minerals. Temperature, pressure, pH, presence or absence of specific ions, ultra-violet light, are all conjured up to fit.

And as to the path to life, we have as many theories as there are molecular types in a cell. Deamer assumes that the organic chemicals that make the necessary structures come from an abiotic environment. But this is an assumption which the hydrothermal vent school disputes. And from that first abiotic chemistry we get metabolism first, genetics first, container first, iron-sulfur world, small molecule world, protein world, lipid world, carbohydrate world and of course RNA world.

Choose your key properties for life, your preferred path, find a location that matches those, and you have a new scenario for life, and one that can match your preferred mechanism better than any other. But it is not an objective choice. For example, Deamer’s choses *fresh* water in part because it uniquely enables self-assembly of fatty acids. But Black and co-workers have found that amino acids can bind to fatty acids (non-covalently) and allow them to form bilayers in the presence of divalent cations [32,33], i.e., in salt water.

And so I nearly put AL down at first glance. In the introduction Deamer contrasts two options—the now-classic cool alkaline serpentinizing seafloor origin and his preferred volcanic hydrothermal field origin—from a list that encompasses dozens if not hundreds. Maybe I am unduly obstreperous, but I felt myself thinking “No, hang on, what about …” before I had got beyond the preface. Which would have been a mistake.

But Deamer makes a more basic, and unstated, assumption, as does most other OOL literature. OOL scenarios usually assume that life occurred in an environment that was fairly predictable, not some fantastic chance occurrence. That itself is not proven. The Principle of Mediocrity (also known as The Copernican Principle) does not apply. If life was a fantastically unlikely, once-in-a-Universe occurrence, we would of necessity be living on the only planet in the entire cosmos that has life; we have to be, because we are alive (this is a version of the Weak Anthropic Principle—the laws of the Universe have to be consistent with us being here to observe it [34]. In this case, the origin of life on Earth has to have happened for us to be here to observe it.) Deamer’s—and everyone else’s—scenarios are trying to explain why conditions on Earth were conducive to OOL. They may not have been. The early Earth, and every other “Earth-like” planet in the cosmos, may have been almost implacably opposed to life, but we made it anyway.

Arguing that specific steps on a proposed path to life are likely does not address this argument. Fatty acids can form multilamellar systems with high probability because thermodynamics favours those systems. But that is only relevant if you think that one of the limiting steps on the path to life is the formation of multilamellar complexes. That depends on your choice of path. For those who follow the serpentenizing alkaline vent model, lamellae are marginal, because redox energy in the vent itself drives polymer formation [35].

This is why I found the experiments posited in Chapter 10 slightly unsatisfying. Despite the quote, Deamer is not trying to solve the problem of how translation appeared. He is looking solely at the earlier step of how to assemble big molecules in water. But, to paraphrase the song, there must be fifty ways to lose some water. Dehydration in lamellae is one way, and has advantages over dry dehydration which Deamer discusses, but many other solutions have been postulated for overcoming “the water problem” [36]; the problem that the forming the polymers of life in dilute solution requires energy (e.g., [35]). None address how you make *specific* polymers from a *mix* of monomer types, nor what happens next. (But do read this yourself—you may be more convinced than I.)

But there is an even bigger problem. We do not even know what life is. We know that we are alive, but we know that we are alive because we define life as what we are. Everything else is conjecture. Many authors have argued that trying formally to define life is both philosophically futile and practically pointless (reviewed in [37,38]), and that we do not need to formally define life to research its origins [39]. Fair enough, but if we are to decide how life originated then we must have some idea of what the minimum requirements are for us to call a system “alive”. As Mix points out [40], we use the term “life” regularly, and we must suppose that we mean something by it, otherwise what are we trying to explain? Hence the “list approach” of listing things life must have, and Deamer lists 14 of them (AL p xvii), but why that list? Are “experiences wet/dry cycles” and “requires genetic information” qualities of the same class? Fish do not experience wet-dry cycles, are they not alive? Weather *is* a wet-dry cycle, does this mean, as Lineweaver has argued [41], that hurricanes cannot objectively be distinguished from life?

This is not pointless sophistry. There is a reason to be more specific in characterizing life (let us not say “defining”), the need for precision in language. Along with most other writers on OOL, Deamer uses “evolve” to mean “change” and to mean “the result of selection for fitter genotypes”, with the implication that one is related to the other. This is seriously misleading. Similarly “replication” can mean production of a new organism from a genetic blueprint (as in “viruses replicate”) or kinetically-determined self-catalysis (as in “crystal defects replicate”). In my (metaphorical) book these are fundamentally different processes; using common language implies they are not, but does not elucidate their similarities or differences. Again, the term ‘Protocell’ is used to mean any liposome-like membrane encapsulating other molecules. In my opinion, a vesicle encapsulating random organic molecules is almost as far from life as the bulk “prebiotic soup” from which it was made. To draw “Protocells → Progenote” in a diagram [11] skips over everything about how that transition happens, i.e., how life originates! And again, polymers that stabilize a vesicle are said to have the function of stabilizing the vesicle, but “function” implies contribution to an aggregate goal of the system that is not implicit in its parts (as the function of a shoe is to protect the foot). Having a property does not imply that something has a function, any more than the gum on my shoe has a function, for all that it has the property of being stably stuck there.

Damer and Deamer, in AL and elsewhere (e.g., [11]) imply that much of this woolly language is analogy or metaphor to illustrate key points, and I afraid that here they push one of my (metaphorical) buttons, because analogies, similies and metaphors make for terrible science. Robbie Burns could sing “My love is like a red, red rose” and not expect his listeners to infer that his belovéd derived her blushing colour from anthocyanins. Instead, we can infer from this factually ridiculous statement something about the singer’s state of mind because we all understand both love and roses. But what if we had no idea what love was? How would we know what aspect of a rose he was talking about? Was Burns celebrating that his love is more closely related to an ear of corn than to himself? When we make similes, analogies and metaphors between things that we do not understand, we risk equating real world properties with rhetorical turns of phrase. At best this is unhelpful, at worst (and this happens often) downright misleading, suggesting that round blobs of chemicals are “like” cells and so in some way are cells.

But studies of the origin of life are not unique in not being able to define or precisely describe their subject matter. Psychiatrists cannot define what mental illness is except as a list of symptoms that we hope we do not have. Researchers in artificial intelligence (AI) cannot agree what intelligence is, except to define it as what we (some of us, probably) have. And yet psychiatry and AI flourish. Why should OOL not do the same?

Because, I suggest, OOL is at least 50 years too early. OOL in 2020 is like AI in 1950. It was missing several critical pieces. We are playing with Toy Domains [42].

After WW-II, there was a huge burst of enthusiasm for computers and their abilities. Science Fiction, popular media and technical researchers alike believed that we were on the brink of generating genuinely intelligent machines, and the only debate was whether they would be evil or not. Stanley Kubrick was considered rather old-fashioned in assuming that the HAL-9000 computer in *2001: A Space Odyssey* could be outwitted by its human crew. Programs were demonstrated that could plan, talk, play games, even diagnose medical conditions [42]. Their real-world application was only a few years away.

In fact, it took 50 years before computers that could genuinely be called ‘smart’ became more than lab toys, and that is because all those early successes were in what are now called Toy Domains. Take SHRDLU, for example, and its application to Blocks World [43]. SHRDLU could take instructions from humans in English and use them to plan the movement of geometric objects in a box. If you said “Put the ball in the large box” it would understand which of the three boxes was the large one, what “ball” and “in” meant, and that it had to remove the pyramid from the large box before it put the ball there. It could understand “Where is the blue pyramid?” and work out which of the two meanings of “put the blue pyramid on the block in the box” was relevant to the “world”. Surely only a small step from there to “Get me to Logan, I’m late for my flight”? No, it is a vast step, because “Blocks World” is a tiny, hyper-simplified version of reality, a toy version of the real world. Winograd realised that, of course, but hoped that the insights gained from SHRDLU would translate into the messier world of real conversation. Largely, they did not. The methods used could not be extended to, say, understanding requests to move objects round a kitchen. It was not just that scale-up was beyond the software, and the hardware, of the day. The algorithms were inherently unsuited to that scaling even when the hardware became available. Scaling from Blocks World to Cab Driver World fundamentally could not work.

In my view, almost all the OOL chemistry that I see is Toy Domain chemistry. It is making single types of biochemicals in a controlled laboratory setting using pure chemicals that might, just might, have been present in trace amounts in a complex mixture of thousands of other chemicals at OOL, under conditions that might have existed and might have persisted long enough, and then stopping the reaction at exactly the right time to maximize the yield of what you want (See [44], especially Chapter 5). It neglects that many of the postulated starting materials are themselves unstable. It neglects that they will react with other chemicals present. It neglects that the intermediates will *all* react with each other, and with the products.

(The occasional comment that some unstable compound is present in meteorites/Titan/comets and so could have been present on early Earth as feedstock for abiotic chemistry is obviously absurd. Stability is determined by environmental conditions, including temperature and presence of other chemicals, including water. Presence of materials on Titan is no more relevant to the proposed inputs to terrestrial scenarios that the presence of DNA on Earth is precedence for life on Mercury. An unstable compound might be a reactive radical or other fleeting intermediate under terrestrial conditions, but that is a different argument.)

Of course OOL chemists understand that 99% pure reagents were not available at OOL. The hope is that by exploring what happens in “clean” chemistry you can gain insight into messier chemistry, and so edge towards more realistic scenarios. Indeed, there is a growing body of work on “messy chemistry”—doing lab chemistry with mixtures and accepting impure products as valid outputs [45,46]. Most researchers, even some working on such chemical schemes, understand that lab chemistry is only a tiny part of the whole problem. But that is not the primary issue. It is a tiny part *solved in an unrealistic way.* Only by a tiny, outside chance can lab reactions of specific reagents, even to give “messy” products, be part of a larger solution. The research does tell us something about chemistry. But it is not something that has much relevance to OOL, because if you carry out lab organic chemistry on anything approaching a plausible pre-biotic aqueous organic soup you never get life. You get tar [44]. Even if you do it in vesicles.

To illustrate, let us accept that organic chemicals accumulate in subaerial ponds and that lamellae would form and that cycles of dehydration would happen and that they would drive dehydration chemistry. What actually would you form if you did *not* start from pure chemicals? It is still controversial that there is any plausible route to make nucleotides in prebiotic conditions [47], so let us look at amino acids, which we know can form abiotically because they are found in meteorites. Deamer mentioned the 15-amino acid antibiotic Gramicidin as an example of a simple peptide that could form pores in his vesicles, and could in principle be formed by dehydration. What would happen if you dehydrated the low molecular weight, water soluble organics actually found in the Murchison meteorite? Even with some optimistic simplifying assumptions, the yield of 15-mer peptides *of any sequence* is ~5 × 10^−10^, and the yield of 15-mer or longer peptides with a pattern of amino acids that might form a pore is <2 × 10^−15^. (See Supplementary Material A). This is a substantial burden of randomness to overcome, even with billions of vesicles. And this does not even start to address chirality.

And it also does not address that pernicious little word “function”.

My argument is not that this chemistry could not happen. It clearly can. My argument is that dehydrating pure monomers in pure lipid, even in hot spring water, is an unrealistic Toy Domain model of how life could have originated. Asking whether it happens only tests the hypothesis that dehydrating pure monomers results in polymers, not the hypothesis that dehydrating mixtures of monomers is a key, limiting step on the path to life.

“Oh, that is unfair!” you will cry. There have been major advances in recent years—work such as Deamer’s and the Russell/Martin school of alkaline serpentizing vent chemistry cited here, as well as many others. And indeed there has been a major advance in the use of the term “major advance” in the OOL literature; 75% of all papers using the phrase “major advance” in the context of origin of life listed in Google Scholar were published after 2011. But what many such advances are is a new scenario—new location, new suggested set of pure reagents to react, a new chain of specific reactions that have be demonstrated, one at a time, in the lab. They are all new Toy Domains.

Deamer admits a lot of this. He points out that, no matter what clever chemistry scheme makes sugars from formaldehyde or adenine from hydrogen cyanide, they form in very low yield and they then break down again under those same conditions. But as AL’s title implies, Deamer has another scale of structure in mind. I am sceptical that focusing on structure gets round those problems, although conceptually blending structure and chemistry seems more promising than focusing solely on either alone, and doing so in a highly parallel combinatorial fashion is also promising. Deamer still needed to start with pure nucleoside phosphates to make a polymeric material that looked “RNA-like”. (Deamer has work in progress to show that it is more “RNA-like” than a random coupling of the monomers; I reserve judgement on how convincing that will be). But it still starts from a pure starting material to make an impure product.

Where do we go from here? Can we get hints how to move beyond this from the history of AI? One hint is sheer scale.

Sufficiently large changes of scale result in qualitative change. The failure of the Toy Domain applications of the 1960s and early 1970s was thought by some to sound the death knell for genuine machine intelligence. For example, could a computer understand natural language *outside* extremely limited, restricted domains? In a famous paper [48], John Searle said “No” to the proposition that any machine without intentionality could understand natural language. He illustrated this with his Chinese Room analogy. Imagine a room that contained only a big book and someone who understands no Chinese, with a narrow slot to the outside world. People write sentences in Chinese on a piece of paper, and push these through a slot into the room. The human looks up the character combinations in the huge book, looks up the appropriate responses, copies them onto another piece of paper, and hands them back out of the slot. To the outside world, people ask the Room questions and get sensible answers. But does the Room understand Chinese? The book obviously does not. The human does not. The room itself is just a box. There is no understanding there at all.

Thirty years on, we see the fallacies in Searle’s analogy. To work, the book would have to be of literally astronomical size to contain every possible sentence in Chinese, and the human be able to move at relativistic speeds to look anything up in it. It is so unrealistic as to be absurd. Since then, the scale of computation has changed by many orders of magnitude, and an equivalent of just such a look-up scheme has become possible. The onboard computers in the Apollo command/service module had a memory of 2 k dynamic memory (and 32 k of ROM). That is 2048 *bytes*. I have been given 10^7^ times that amount of memory in a USB flash drive as a free marketing giveaway. Now we understand that datasets bigger than the entire of digitized knowledge in 1980 and processors billions of times more capable can indeed execute what amounts to a Chinese Room translation, and use the resulting “understanding” to direct their behaviour. More, this combination of huge datasets and complex algorithms shows disturbingly powerful insights into our wants, needs and likely desires. Sheer power and parallelization has solved the problem.

(Does Facebook ‘understand’ my political opinions, and direct advertising at me accordingly? Alan Turing famously said that it does not matter [49]. If it is behaving intelligently in the general world, as opposed to in a Toy Domain, it is in effect intelligent.)

So raw power can solve some problems, and Deamer recognises this. In the final chapter of FL, and to a less bold degree in chapter 7 of AL, Deamer describes his ‘prebiotic Earth in a box’ experiments that try to move away from simplistic, conventional chemistry. He wants complicated experiments that chemists would eschew because they are hard to analyse. He wants highly parallel combinatorial chemistry, to have a chance of finding the tiny corner of physicochemical space in which complex structure develops. His vision of an OOL box described in FL might be seen in retrospect as naïve as SHRDLU, but it would be a start *if* it could be parallelized to the extent that chemistry has been parallelized in high-throughput discovery technology for materials [50] or drugs [51]. This is research devoid of a specific model for life, which has its place in biochemical research [52]. Ironically, current OOL is entirely hypothesis driven, there is almost no data beyond spot checks that specific chemical reactions do, indeed, happen in the lab. (Much of Chapter 10 is this sort of test. Cycling lipid/monomer systems between vesicle and lamellar phases under conditions where dehydration polymers form will indeed show that polymers form. But attributing “function” to them is teleology of a different kind.)

I think we need to go beyond this, and here Deamer is oddly muted. We need new ideas, and not just yet another contrived scenario how this or that reaction could happen on this or that mineral, not even a refocusing on the chemistry of life on self-organization rather than individual chemical reactions. We need fundamentally new ways of looking at life and its origin, and we do not have them, not even close.

To take another analogy, OOL today is (in my opinion) in the position of 19th century astronomers theorizing on why the Sun shines. Their theories were imaginative, thoughtful, authoritative, quantitative, and completely wrong (See Supplementary B for a summary of what they were.) Without knowledge (radioactivity, fusion, atomic structure), and concepts (mass-energy equivalence) unknown to 19th Century science, the task was impossible. We smile condescendingly at their ideas today, and then earnestly postulate that life originated through imidazole-catalysed polymerization of nucleoside precursors in intertidal pools. We should imagine our successor’s smiles.

What new ideas? I do not know! I can speculate that we need to re-examine the concept of causality in biology, a notion that is often invoked and rarely works. Just one illustration of this—much of modern drug discovery is the based on the idea of a molecular “target” that is causal in a disease pathology. Despite hundreds of millions of dollars per project, that approach has a <5% success rate [53], and the more we discover about supposedly causal networks in the cell, the more the success rate declines [54]). With Sydney Brenner and Denis Noble, I suspect that biological systems defy the sort of causality implicit in diagrams of boxes and arrows labelled “replication” and “metabolism” [55]. [38] provides a recent, deep and informed discussion of this, and how ancient ideas of causality going back to Aristotle are still replicated in today’s origin hypotheses. It just might be time to admit that Aristotle is not a good basis for 21st century science.

We probably need a fundamentally different language for describing how cells work (see [56] for a highly entertaining commentary on this). Deamer makes a foray into this in Chapter 8, with a nomenclature for prebiotic simulations, a nomenclature which predictably predicts that his experimental approach includes the largest set of factors, and hence is implied to be the best simulation of abiogenesis. I was unconvinced. He assumes his 18 factors are necessary and sufficient to generate life-like systems. The very existence of the diversity of opinion in OOL research suggests that we are not close to a consensus on that. But at least he has tried, and given others new substrate for argument and thought.

And in my opinion, we need that language to be precise and defined, not metaphorical. When we say “replicate” we need to know *exactly* what we mean by that. For what it is worth I think the key difference between life and non-life lies in coded information [57]. But these are words. What *precisely* do they mean? My (and others’) inability to define this in chemically testable terms is one on the major roadblocks in our understanding of the emergence of life that AL hardly mentions; how translation arises (and the capacity for evolution as a necessary consequence of translation [37]).

There is also a lot of excitement about “systems chemistry” and “autocatalytic” systems, catalysed mainly by Stuart Kauffman [58]. Kaufmann postulates that a sufficiently diverse collection of reactive and catalytic molecules would undergo a phase transition and become self-propagating, autocatalytic, i.e., life-like. But what does “diverse” mean? Atmospheric photochemical networks have nearly as many components and more reactions than central metabolism [59]. Does this mean the atmosphere is alive? Specificity and accuracy need to be included; what biochemicals do *not do* is at least as important we what they do [60]. But this depends on the chemistry involved. Just saying that A catalyses the reaction of B with C does not say what A does to D thru Z, or what D thru Z do to A. Even within the biochemical networks of established life, random chemistry occurs and degrades the components of metabolism (e.g., the reaction of amines with sugars, amino acid side-chains with each other etc. [61]). Any sufficiently complex set of reactive and catalytic molecules is, in fact, Benner’s tar [44]. We need something more.

And we hear every now and again about “New Physics” [58], a term growing like bindweed from the intellectual rootstock to Schroedinger’s execrable book. I do not believe that we need new physics any more that 19th Century astronomers needed new orbital mechanics (unless you define physics as “any science that is not today’s chemistry or biology”). We need new something, but lack the words to even describe what we need. It is not physics. It is not chemistry. But whatever it is, it must talk to physics, chemistry, biology, and to the real world that Deamer describes eloquently and concisely, and it must be testable, as Deamer makes clear in Chapter 10 and in [9]. Grand abstractions or unworkable networks of hypothetical chemistry will not do.

And for this, last reason, grounding in the world of the real, Deamer’s vision is valuable. Read the book. Disagree with it, or with me, or both. Learn from it. And then go away and think.

## Supplementary Materials

The following are available online at https://www.mdpi.com/2075-1729/10/2/18/s1, Supplementary text A: Description of model of dehydration polymerization of small molecules present in Murchison meteorite. (Table S1: water-soluble molecules in Murchison. Figure S1: model logic, Figure S2: results of model). Supplementary text B: short summary of late Victorian histories of why the Sun shines.
