# Supplementary material for: Getting Beyond the Toy Domain. Meditations on David Deamer’s “Assembling Life”
Source: Life (Basel). 2020 Feb 18;10(2):18. doi: 10.3390/life10020018 (PMC7175206; doi:10.3390/life10020018)
Supplement: Supplementary file 1 [file life-10-00018-s001.pdf]

Getting beyond the toy domain.

Meditations on David Deamer's "Assembling Life"

William Bains

Supplementary A: random polymers are not peptides

If you made a random polymer from abiotic chemicals, what actually would be made? I did a simple model of this as follows. I take the Murchison meteorite as an example of the water-soluble monomers that can be formed abiotically in aqueously processed rock [1]. Over 70 amino acids have been identified in Murchison [2], and this is often taken as an example of how biochemical can be made abiotically.

The condensation of such abiotic amino acids is usually depicted as in Figure A1-A. However the reality is that many other monomers will be present in the "soup", and the reaction is more likely to be as in Figure A1-B. So what would form if you dehydrated the Murchison soluble organics? To condense together with the elimination of water, monomers must have an acid group (carboxylic, phosphonic or sulphonic acids), a condensable group (amine or alcohol), or both. I ignore acid anhydrides for this purpose, as they are more liable to hydrolysis than esters. I assume that condensation is kinetically driven purely by abundance of the monomers. The actual reaction rates will be an unknowable function of thermodynamics, water activity, ionization state, and the specific reaction kinetic constants between individual molecules. I also assume that polymerization does not affect the concentration of monomers in the pool; for relatively inefficient abiotic condensation, this is realistic. Lastly, I assume that condensation is purely end-to end, ignoring side-chain condensation and consequently branching, and further that an amide bond, once formed, does not react further (to form acylated amides, for example). All these assumptions favour formation of simple linear "peptide-like" polymers.

The table of monomers and their relative concentrations is given below. There are six classes of monomers with respect to their ability to condense. Rather than try to model specific polymers, I model the condensation of classes of molecule. Thus any molecule of class APC (alpha amino acids) can condense with any molecule of class AEC (hydroxyl acids) either to form an APC-AEC dimer or an AEC-APC dimer. The chances of AEC condensing with an APC are proportional to the relative amount of AEC. In that case, Class XA (monocarboxylic, phosphonic and sulphonic acids) and XC (amines and alcohols) are 'chain terminators' in that once they have condensed to form an amide they do not have an additional functional group to react with an additional monomer.

From the relative abundances in Table A1, we might expect the most common dipeptide to be glycylglycine. Analysis of dipeptides in Murchison finds 4pmol/g of glycylglycine, 23 pmol of cyclized glycylglycine, and no detectable amount of any other dipeptide or cyclized dipeptide [3], which is consistent with the idea that dehydration forms random oligomers based on monomer stoichiometry.

Analytical calculation of the probability of the many combinations of monomers is impractical, and so I simulated polymerization as follows. The algorithm is seeded with a 'polymer' of one monomer unit, selected at random from the pool of monomers proportionally to their concentration. At each step, we examine both ends of the growing chain. If it is an acid, we select a molecule with a

condensable group with a probability proportional to its concentration, and join that to the end. If the end is a condensable end, we select a molecule that has an acid group with a probability proportional to its concentration, and join that to the condensable end. This generates a new, longer polymer. (Note that the 'direction' of the polymer can reverse – a dicarboxylic acid can join to two amines, for example, like nylon). We continue this until the polymer has no reactable ends. (Gramicidin is an example of this, 'capped' by formate at the amino terminus and ethanolamine at the carboxyl terminus [4])

The results of some simulations are shown in Figure A2. Plotted are the length of oligomer formed. As we assume condensation, there are no oligomers of N=1. N=2 is the condensation of an acid with an alcohol or amide to form a simple ester or amide respectively – these dominate the products. Longer products are, as expected, exponentially less likely.

It has been argued that alpha amino acid polymers are uniquely suited to forming the catalytic units of life, because of backbone stereochemistry [5, 6]. The algorithm therefore also counts the number of chains made that are composed solely of alpha amino acids (not counting the chain terminating groups), and for comparison the number of polymers made solely with amide groups (i.e. condensation of acids with alpha, beta or gamma amino acids), and solely of esters[1]. The results are smooth exponentials, as expected: extrapolation the chance of making a 15-mer, the same size as Gramicidin, an antibiotic peptide cited by [7] as an example of a prototype membrane-spanning pore peptide required for water and solute balance in vesicles, is ~$5 \bullet 10^{-10}$. If the gramicidin-like peptides needs a specific pattern of amino acids to function, the chances are smaller. We can estimate the chances of forming a *functional* peptide of 15 amino acids as follows. We assume the peptide needs a pattern of hydrophobic and hydrophilic amino acids along the chain, spaced by any small amino acid. If we assume $^1/_3$ of the amino acids need to be hydrophilic, $^1/_3$ hydrophobic and the rest can be any amino acid, then the fraction of peptides that are 15-mers with the "functional" pattern of amino acids is ~$1.5 \bullet 10^{-15}$

The underlying reason for the low yield of peptides is the relatively low abundance of amino acids. Reports of discovery of >70 amino acids do not put the tiny traces of these compounds found into context among a much larger amount of other organics. To generate a better yield of peptides we need to concentrate the amino acids. Figure A3 illustrates the result of increasing the abundance of amino acids while keeping the abundances of all other species the same (i.e. concentrating the amino acids). This shows that to have ~1:1000000 chance of making a gramicidin-like peptide by random condensation, you need to concentrate the amino acid fraction of Murchison selectively over all the other components by 100-fold or more.

This is a simplistic model, and as such is justifiably relegated to an appendix. However it illustrates that the issue in OOL research is not just 'can we make it' but also 'can we stop making everything else'.

---

[1] In fact it counts the chains that do not have amide bonds in as ones that are all=ester, and hence two-monomer 'polymers' are counted as esters. Similarly, all 2-monomer chains are counted as amides and peptides.

Table A1

Abundances of soluble components from the Murchison meteorite. Data from [1, 3, 8-20]. Abundances for chiral compounds were pooled, as chirality was not considered in this calculation. The lowest abundance recorded is much lower for amino acids than for other classes, but this has little effect on the total abundance of the monomer class. Some acids and both amino- and hydroxy-acids; they are classified according to the group nearest the carboxylate group.

Compound	mwt	SMILES	Abundance (nmol/g)	Class of monomer	Total abundance of monomer class
oxalic acid	90.034	O=C(O)C(O)=O	2100	AEA (dicarboxylic acids)	2466
succinic acid	118.088	O=C(O)CCC(O)=O	97		
malonic acid	104.061	O=C(O)CC(O)=O	96		
2-methylsuccinic acid	132.115	O=C(O)C(C)CC(O)=O	48		
glutaric acid	132.115	O=C(O)CCCC(O)=O	24		
citraconic acid	130.099	C/C(C(O)=O)=C/C(O)=O	19		
2-methylglutaric acid	146.142	O=C(O)C(C)CCC(O)=O	15		
ethylsuccinic acid	146.142	O=C(O)C(CC)CC(O)=O	14		
methylmalonic acid	118.088	O=C(O)C(C)C(O)=O	9.2		
2,2-dimethylsuccinic acid	146.142	O=C(O)C(C)(C)CC(O)=O	8.8		
adipic acid	146.142	O=C(O)CCCCC(O)=O	5.8		
2-methyladipic acid	160.169	O=C(O)C(C)CCCC(O)=O	5.4		
3-methylglutaric acid	146.142	O=C(O)CC(C)CC(O)=O	4.7		
(meso)-2,3-dimethylsuccinic acid	146.142	O=C(O)C(C)C(C)C(O)=O	3.26		
phthalic acid	166.132	O=C(O)C1=CC=CC=C1C(O)=O	3.2		
azelic acid	188.223	O=C(O)CCCCCCCC(O)=O	3.2		
3-ethylglutaric acid	160.169	O=C(O)CC(CC)CC(O)=O	2.9		
ethylmalonic acid	132.115	O=C(O)C(CC)C(O)=O	2.3		
3-methyladipic acid	160.169	O=C(O)CC(C)CCC(O)=O	1.9		
suberic acid	174.196	O=C(O)CCCCCCC(O)=O	1.7		
maleic acid	116.072	O=C(O)/C=C\C(O)=O	1		

lactic acid	90.078	OC(C)C(O)=O	65.8	AEC (hydroxy acids)	389
glycolic acid	76.051	OCC(O)=O	65		
2-hydroxycaprioc acid	132.159	CCCCC(O)C(O)=O	65		
2-hydroxyglutaric acid	148.114	O=C(O)C(O)CCC(O)=O	54.45		
2-hydroxy-3-methylbutyric acid	118.132	CC(C)C(O)C(O)=O	36.9		
2-hydroxyisobutyric acid	104.105	CC(C)(O)C(O)=O	30		
2-hydroxybutyric acid	104.105	CCC(O)C(O)=O	19		
3-hydroxy-isobutyric acid	104.105	CC(CO)C(O)=O	18.3		
2-hydroxy-2-methylbutyric acid	118.132	CCC(C)(O)C(O)=O	12.4		
3-hydroxybutyric acid	104.105	CC(O)CC(O)=O	10.4		
2-hydroxyvaleric acid	118.132	CCCC(O)C(O)=O	4.7		
2-hydroxy-2-ethylvaleric acid	146.186	CCCC(CC)(O)C(O)=O	3.5		
2-hydroxy-2-methylvaleric acid	132.159	CCCC(C)(O)C(O)=O	3		
4-amino-2-hydroxybutyric acid	119.12	O=C(O)C(O)CCN	0.235		
Glycine	75.067	NCC(O)=O	33.5	APC (alpha amino acids)	190
2-amino-2,3-dimethylpentanoic acid	145.202	CCC(C)C(C)(N)C(O)=O	27		
Alanine	89.094	NC(C)C(O)=O	17.94		
Glutamic acid	147.13	NC(CCC(O)=O)C(O)=O	11.27		
a-aminobutyric acid	103.121	CCC(N)C(O)=O	8.58		
Proline	115.132	O=C(O)C1NCCC1	8.02		
2-amino-2,3-dimethylbutyric acid	131.175	CC(C)C(C)(N)C(O)=O	8		
a-amino-isobutyric acid	103.121	CC(C)(N)C(O)=O	7.49		
Aspartic acid	133.103	NC(CC(O)=O)C(O)=O	6.32		
serine	105.093	NC(CO)C(O)=O	6.02		
2-methylnorvaline	131.175	N[C@](CCC)(C)C(O)=O	6		
2-amino-2-ethylbutyric acid	131.175	CCC(CC)(N)C(O)=O	6		
Valine	117.148	NC(C(C)C)C(O)=O	5.42		
2-amino-2,4-dimethylpentanoic acid	145.202	CC(C)CC(C)(N)C(O)=O	5.3		
isovaline	117.148	N[C@@](C(O)=O)(CC)C	5.28		
sarcosine	89.094	CNCC(O)=O	3.36		
2-amino-2-ethylpentanoic acid	145.202	CCCC(CC)(N)C(O)=O	3		
2-amino-2-methylhexanoic acid	145.202	CCCCC(C)(N)C(O)=O	2.8		
2-amino-3,3-simethylpentanoic acid	145.202	CCC(C)(C)C(N)C(O)=O	2.7		
threonine	119.12	NC(C(O)C)C(O)=O	2.61		
leucine	131.175	NC(CC(C)C)C(O)=O	2.07		

norleucine	131.175	NC(CCCC)C(O)=O	2		
isoleucine	131.175	NC(C(C)CC)C(O)=O	1.88		
2-amino-3,4-dimethylpentanoic acid	145.202	CC(C)C(C)C(N)C(O)=O	1.6		
alpha-aminoadipic acid	161.157	O=C(O)C(N)CCCC(O)=O	1.446		
2-amino-3-methylhexanoic acid	145.202	CCCC(C)C(N)C(O)=O	1.2		
2,3-diaminopropanoic acid	104.109	O=C(O)C(N)CN	0.957		
norvaline	117.148	NC(CCC)C(O)=O	0.778		
2,3-diaminobutanoic acid	118.136	CC(N)C(N)C(O)=O	0.761		
alpha-methylserine	119.12	N[C@](CO)(C)C(O)=O	0.663		
homoserine	119.12	NC(CCO)C(O)=O	0.175		
gamma-aminobutyric acid	103.121	O=C(O)CCCN	11.3	AQC	47
beta-alanine	89.094	NCCC(O)=O	7.32		
5-amino-pentanoic acid	117.148	O=C(O)CCCCN	5.52		
4-amino-3-methylbutanoic acid	117.148	O=C(O)CC(C)CN	5		
beta-amino-isobutyric acid	103.121	CC(CN)C(O)=O	3.81		
4-aminopentanoic acid	117.148	CC(N)CCC(O)=O	2.55		
4-amino-2-methylbutanoic acid	117.148	O=C(O)C(C)CCN	2.24		
3-amino-2,2-dimethylpropanoic acid	117.148	O=C(O)C(C)(C)CN	2		
n-methylalanine	103.121	CC(C(O)=O)NC	0.9		
6-aminohexanoic acid	131.175	O=C(O)CCCCCN	0.8		
3-amino-2-ethylpropanoic acid	117.148	O=C(O)C(CC)CN	0.6		
2,4-diaminobutanoic acid	118.136	O=C(O)C(N)CCN	0.52		
3-amino-2-(aminomethyl)propanoic acid	118.136	O=C(O)C(CN)CN	0.411		
beta-(aminomethyl)-succinic acid	147.13	O=C(O)CC(CN)C(O)=O	0.387		
3-amino-2-(hydroxymethyl)-propanoic acid	119.12	O=C(O)C(CO)CN	0.277		
4-amino-3-hydroxybutyric acid	119.12	O=C(O)CC(O)CN	0.277		
4-amino-3-(aminomethyl)butanoic acid	132.163	O=C(O)CC(CN)CN	0.244		
beta-homoserine	119.12	NC(CO)CC(O)=O	0.101		
beta-amino-n-butyric acid	103.121	CC(N)CC(O)=O	2.840		
2,5-diaminopyrrole	97.121	NC1=CC=C(N)N1	0.407	CQC (diamines)	0.407
acetic acid	60.052	CC(O)=O	4956	XA (monocarboxylic acids)	20734
n-propanoic acid	74.079	CCC(O)=O	3662		
benzoic acid	122.123	O=C(O)C1=CC=CC=C1	2325		
2-methylpropanoic acid	88.106	CC(C)C(O)=O	2014		
n-butanoic acid	88.106	CCCC(O)=O	1843		

n-pentanoic acid	102.133	CCCCC(O)=O	1404		
2-methylbutanoic acid	102.133	CCC(C)C(O)=O	749		
2-methylpentanoic acid	116.16	CCCC(C)C(O)=O	505		
n-heptanoic acid	130.187	CCCCCCC(O)=O	468		
3-methylpentanoic acid	116.16	CCC(C)CC(O)=O	460		
n-hexanoic acid	116.16	CCCCCC(O)=O	409		
ethylsulfonic acid	110.127	CCS(=O)(O)=O	380		
2-methylhexanoic acid	130.187	CCCCC(C)C(O)=O	343		
methylsulfonic acid	96.1	CS(=O)(O)=O	271		
2-ethylbutanoic acid	116.16	CCC(CC)C(O)=O	212		
n-nonanoic acid	158.241	CCCCCCCCC(O)=O	209		
isopropylsulfonic acid	124.154	CC(S(=O)(O)=O)C	190		
n-octanoic acid	144.214	CCCCCCCC(O)=O	181		
2-ethylhexanoic acid	144.214	CCCCC(CC)C(O)=O	84		
n-propylsulfonic acid	124.154	CCCS(=O)(O)=O	54		
Methyphosphonic acid	96.021	CP(O)(O)=O	9		
Ethylphosphonic acid	110.049	CCP(O)(O)=O	6		
methanol	32.042	CO	156	XC (alcohols and amines)	348
ethanol	46.069	CCO	65.12		
Methylamine	31.058	NC	49		
isopropanol	60.096	CC(O)C	33.28		
Ethylamine	45.085	NCC	14		
butanol	74.123	CCCCO	13.49		
isopropylamine	59.112	NC(C)C	9		
n-propylamine	59.112	NCCC	5		
tert-butylamine	73.139	NC(C)(C)C	3		

FIGURE A1

Cartoon of text-book and realistic condensation chemistry

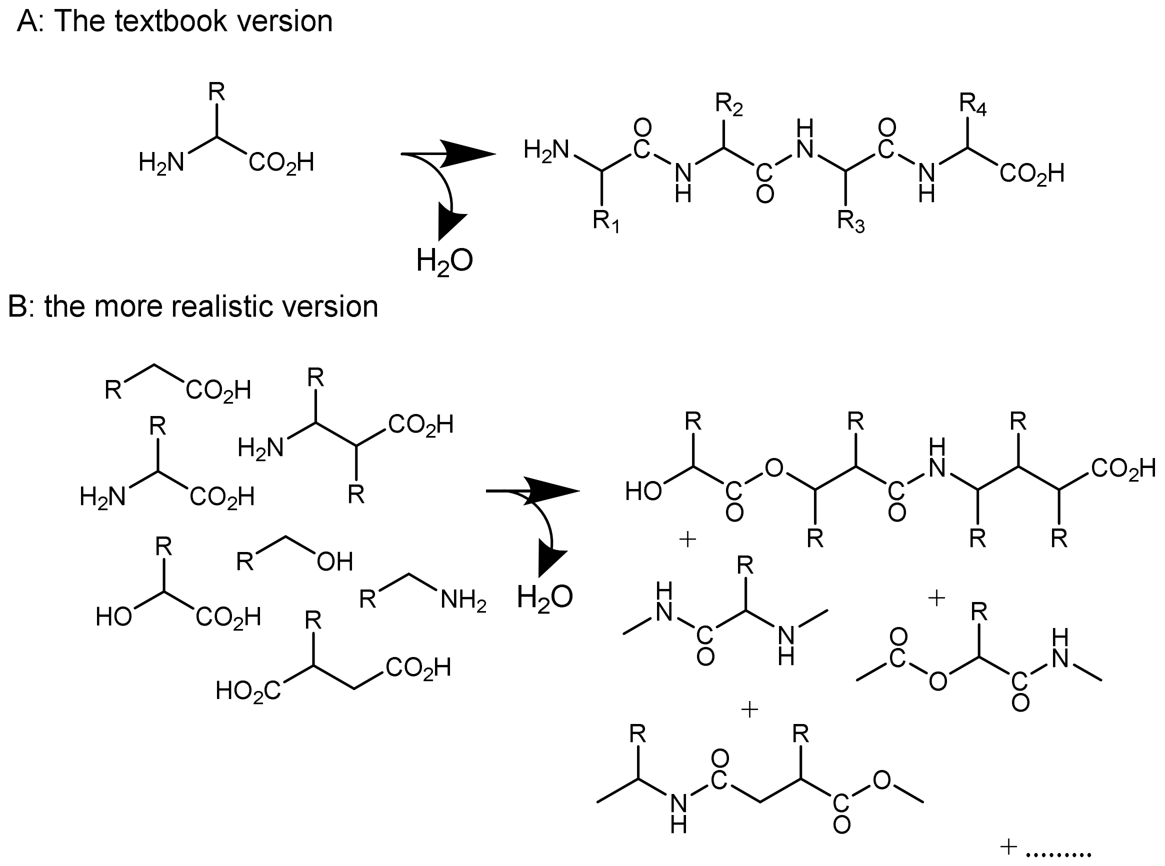

Figure A2

A

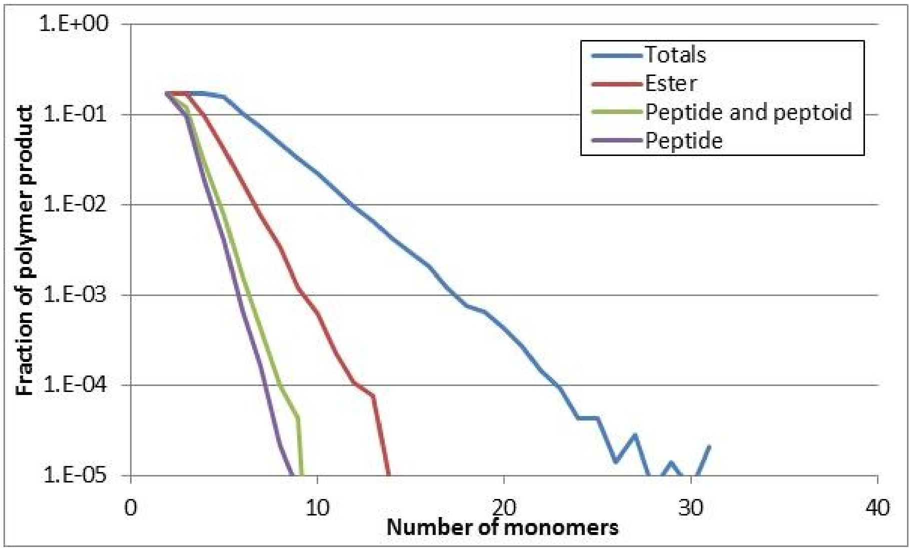

Fraction of 'polymerization' products from the model (Y axis) as a function of the number of monomers in the polymer (X axis). Shown are the total polymer amounts, and the fraction that have exclusively peptide, amide or ester linkages. The erratic lines below ~10-4 are due to small number counting errors.

B.

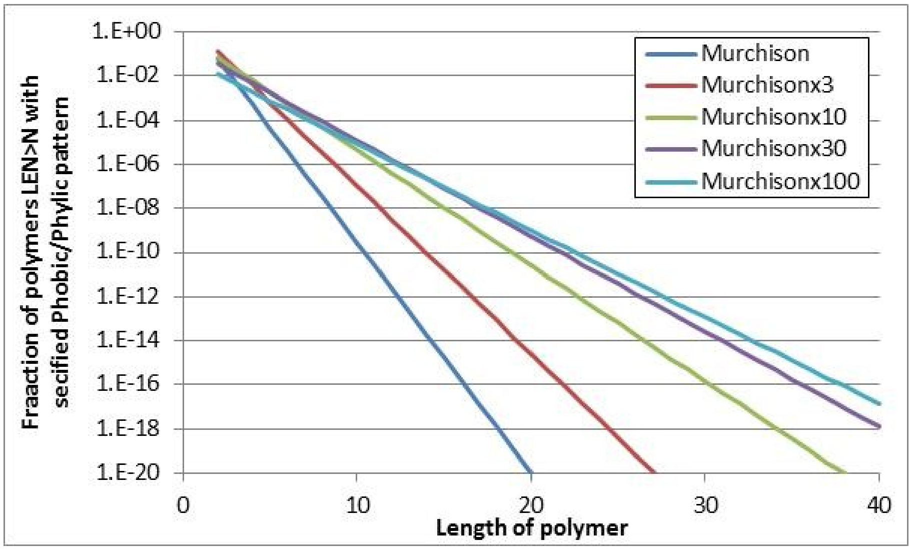

Fraction of the total 'polymer' product in the model that a) are peptides and b) have $^{1}/_{3}$ of amino acids as hydrophilic and $^{1}/_{3}$ as hydrophobic in a specified pattern (Y axis) .vs polymer length (X axis). Curves show calculations for amino acid abundances in Murchison, and for abundances where the alpha amino acids are 3, 10,30 and 100 times as abundant (all other abundances being held constant) as is found in Murchison.

Getting beyond the toy domain.

Meditations on David Deamer's "Assembling Life"

William Bains

Supplementary B: why does the Sun shine?

By about 1840 it was clear that the Earth was millions of years old [21], and the Sun had been shining for as long as the Earth had been around. Until this time, the radiance of the Sun was taken as a given, it just was. But scientific enquiry was finding mechanisms for every aspect of the known universe, and so the source of the Sun's heat became a problem for scientific debate. See [22-24] for post-Victorian reviews of this.

The obvious solution was that the Sun was on fire. 19th Century calorimetry estimated the rate of heat production from the Sun quite accurately. If the Sun's energy was radiated uniformly into space, then if the Sun was entirely made of high grade anthracite coal it would burn for less than 6000 years, so that could not be an explanation. A range of other ideas were floated, including that the Sun's heat was directed solely towards the planets, that it was caused by impact of very high-speed objects from interstellar space, or that it was the result of release of latent heat of condensation or crystallization of some unknown substance in the Sun, but these could be discounted on quantitative grounds [25].

Two major theories remained – that heat was generated from the infall of meteoritic material (e.g [26, 27]), or that it was the energy of gravitational contraction of the Sun [28]. Both derived energy from gravity.

The problem with the meteoritic infall theory was that it should also apply to the planets, which should be at least red hot, and that the Sun's mass should have increased several percent in historical times, resulting in changes in the length of the year; neither were seen.

The problem with the contraction theory was that it could only release heat at roughly the modern rate for the past 10 – 20 million years [25]. That was a problem for geologists, who thought the Earth at least 300-500 million years old. Their argument lay on the rate of accumulation and erosion of sedimentary rock, and if sedimentary rock was accumulating then rain had to be falling, rivers running, and that required sunshine to power weather cycles. Some physicists, notably Lord Kelvin, disagreed, calculating that the Earth was no more than 10-20 million years old, based on the rate of cooling from an initially liquid state, and on the observed temperature gradient in mines. But the weight of evidence on the geologists' side was winning the argument.

So neither dominant theory explained the facts, and there was no better theory. There the problem sat at the end of the 19th Century, and was sufficiently intractable that many popular astronomy books simply did not mention it at all (e.g. the otherwise detailed [29, 30]).

There were a range of outré ideas, and in one or two, in retrospect, we can see some glimmer of ideas that almost seem prophetic, as in this quote from [25]

> "The suggestion made many years ago by Professor Clarke, of Cincinatti, that the sun's radiation may be due in part due to the combination of the really elementary constituents of

> his [2] mass into the substances which we regard as elementary, is open to the objection of being as yet unsupported by trustworthy evidence respecting the really compound nature of the so-called elements." (p336)

But in reality this did not presage the discovery of nuclei, nuclear binding energy, mass-energy equivalence and hydrogen fusion – it was an incorrect extension of chemical ideas, and was speculation, at best conjecture, rather like much OOL research today. And we note that equally speculative ideas were spectacularly wrong, such as the argument in the same book that the speed of gravity had to be very much greater than the speed of light (if it were not, the delay in gravity getting from the Sun to the Earth would mean that Earth was pulled towards the apparent position of the Sun, the position it in fact occupied 8 minutes ago rather than its real position, just as the light we see represents the Sun's position 8 minutes ago and not it's position now. The accumulating difference between the 'true' vector and the offset vector would mean that the Earth would fall into the Sun in a few years. I leave finding the fallacy in this as an exercise for the reader.)

The glimmer of the real source of the Sun's energy came in 1905 with $E=MC^2$, and it came not from astronomers but from a patent clerk pondering Maxwell's equations. We must wonder where the equivalent conceptual leap in OOL will come from.

---

[2] 19th Century astronomy books almost always referred to the Sun as 'him' not 'it'.

References

1. Cronin, John R., and Sherwood Chang. "Organic Matter in Meteorites: Molecular and Isotopic Analyses of the Murchison Meteorite." In *The Chemistry of Life's Origins*, edited by J. M. Greenberg, C. X. Mendoza-Gómez and V. Pironello, 209-58. Dordrecht: Springer Netherlands, 1993.
2. Cronin, JR, GW Cooper, and S Pizzarello. "Characteristics and Formation of Amino Acids and Hydroxy Acids of the Murchison Meteorite." *Advances in Space Research* 15, no. 3 (1995): 91-97.
3. Shimoyama, Akira, and Ryo Ogasawara. "Dipeptides and Diketopiperazines in the Yamato-791198 and Murchison Carbonaceous Chondrites." *Origins of Life and Evolution of Biospheres* 32 (2002): 165 - 79.
4. Wallace, B A. "Gramicidin Channels and Pores." *Annual Review of Biophysics and Biophysical Chemistry* 19, no. 1 (1990): 127-57.
5. Fischer, Gunter. "Chemical Aspects of Peptide Bond Isomerisation." *Chemical Society Reviews* 29, no. 2 (2000): 119-27.
6. Choudhary, Amit, and Ronald T Raines. "An Evaluation of Peptide-Bond Isosteres." *ChemBioChem* 12, no. 12 (2011): 1801-07.
7. Deamer, David. *Assembling Life: How Can Life Begin on Earth and Other Habitable Planets?* New York, NY, USA: Oxford University Press, 2019.
8. Cronin, J. R., W. E. Gandy, and S. Pizzarello. "Amino Acids of the Murchison Meteorite. I. Six Carbon Acyclic Primary Alpha-Amino Alkanoic Acids " *Journal of Molecular Evolution* 17, no. 5 (1981): 265-72.
9. Cronin, John R., Sandra Pizzarello, and George U. Yuen. "Amino Acids of the Murchison Meteorite: Ii. Five Carbon Acyclic Primary Β-, Γ-, and Δ-Amino Alkanoic Acids." *Geochimica et Cosmochimica Acta* 49, no. 11 (1985): 2259-65.
10. Cooper, George W, Wilfred M Onwo, and John R Cronin. "Alkyl Phosphonic Acids and Sulfonic Acids in the Murchison Meteorite." *Geochimica et Cosmochimica Acta* 56, no. 11 (1992): 4109-15.
11. Cronin, John R, and Sandra Pizzarello. "Amino Acids of the Murchison Meteorite. Iii. Seven Carbon Acyclic Primary Α-Amino Alkanoic Acids1." *Geochimica et Cosmochimica Acta* 50, no. 11 (1986): 2419-27.
12. Jungclaus, G., J. R. Cronin, James Moor, and G. Yuen. "Aliphatic Amines in the Murchison Meteorite." *Nature* 261, no. 5556 (1976): 126.
13. Kvenvolden, Keith A, James G Lawless, and Cyril Ponnamperuma. "Nonprotein Amino Acids in the Murchison Meteorite." *Proceedings of the National Academy of Sciences* 68, no. 2 (1971): 486-90.
14. Lawless, James G, and George U Yuen. "Quantification of Monocarboxylic Acids in the Murchison Carbonaceous Meteorite." *Nature* 282, no. 5737 (1979): 396.
15. Shimoyama, Akira, and Ryusuke Shigematsu. "Dicarboxylic Acids in the Murchison and Yamato-791198 Carbonaceous Chondrites." *Chemistry letters* 23, no. 3 (1994): 523-26.
16. Aponte, José C., Rafael Tarozo, Marcelo R. Alexandre, Conel M. O'D Alexander, Steven B. Charnley, Christian Hallmann, Roger E. Summons, and Yongsong Huang. "Chirality of Meteoritic Free and Iom-Derived Monocarboxylic Acids and Implications for Prebiotic Organic Synthesis." *Geochimica et Cosmochimica Acta* 131 (2014): 1-12.
17. Meierhenrich, Uwe J, Guillermo M Muñoz Caro, Jan Hendrik Bredehöft, Elmar K Jessberger, and Wolfram H-P Thiemann. "Identification of Diamino Acids in the Murchison Meteorite." *Proceedings of the National Academy of Sciences* 101, no. 25 (2004): 9182-86.

18. Pizzarello, Sandra, Yi Wang, and Galina M Chaban. "A Comparative Study of the Hydroxy Acids from the Murchison, Gra 95229 and Lap 02342 Meteorites." *Geochimica et Cosmochimica Acta* 74, no. 21 (2010): 6206-17.
19. Jungclaus, GA, GU Yuen, CB Moore, and JG Lawless. "Evidence for the Presence of Low Molecular Weight Alcohols and Carbonyl Compounds in the Murchison Meteorite." *Meteoritics* 11 (1976): 231-37.
20. Koga, Toshiki, and Hiroshi Naraoka. "A New Family of Extraterrestrial Amino Acids in the Murchison Meteorite." *Scientific reports* 7, no. 1 (2017): 636.
21. Dalrymple, G Brent. "The Age of the Earth in the Twentieth Century: A Problem (Mostly) Solved." *Geological Society, London, Special Publications* 190, no. 1 (2001): 205-21.
22. Armitage, Angus. *A Century of Astronomy* London, UK: Samson Low, 1950.
23. Pannekoep, A. *A History of Astronomy*. London, UK: George Allan and Unwin, 1961.
24. Jeans, James. *The Universa around Us*. Cambridge, UK: Cambridge University Press, 1930.
25. Proctor, Richard A., and A. Cowper Ranyard. *Old and New Astronomy*. London, UK: Longamn Green and Co, 1892.
26. Waterston, J. J. "On Certain Inductions with Respect to the Heat Engendered by the Possible Fall of a Meteor into the Sun; and on a Mode of Deducing the Absolute Temperature of the Solar Surface from Thermometric Observation." *The London, Edinburgh, and Dublin Philosophical Magazine and Journal of Science* 19, no. 128 (1860): 338-43.
27. Mayer, J. R. *Beiträge Zur Dynamic Des Himmels in Populärer Darstellung* Heilbron: Verlag von Johann Ulrich Landherr (see https://babel.hathitrust.org/cgi/pt?id=chi.085969603), 1848.
28. Helmholtz, H von. "Über Die Wechselwirkung Der Naturkräfte Und Die Darauf Bezüglichen Neuesten Ermittlungen Der Physik (1854)." *ders., Vorträge und Reden* 1 (1902): 48-83.
29. Clerke, Agnes M. *A Popular History of Astronomy Druing the 19th Century*. London, UK: Adam and Charles Black Ltd, 1902.
30. Stawell, Robert. *The Story of the Heavens*. London, UK: Cassell and Company, 1901.
